# Unexpected Splenic and Retroperitoneal Uptake on Technetium-99m Pyrophosphate (Tc-99m PYP) Imaging: Expanding the Spectrum of Extracardiac Findings

**DOI:** 10.7759/cureus.107874

**Published:** 2026-04-28

**Authors:** Stephanie Crass, Marc T Zughaib, Souheil Saba

**Affiliations:** 1 Cardiology, Henry Ford Providence Hospital, Southfield, USA

**Keywords:** amyloidosis, attr, attr cardiac amyloidosis, cardiac amyloidosis, spect, tc-99m

## Abstract

The diagnosis of transthyretin cardiac amyloidosis (ATTR-CA) remains challenging. Extracardiac uptake of technetium-99m pyrophosphate (Tc-99m PYP) is less understood and may provide insights into other underlying conditions. This report presents a rare case of extracardiac Tc-99m PYP uptake in a patient with retroperitoneal hemorrhage.

A 55-year-old female patient with no history of coagulopathy or anticoagulant use presented with left flank and abdominal pain. Contrast tomography imaging of the abdomen and pelvis revealed a large left retroperitoneal hematoma of unknown etiology. Consequently, a Tc-99m PYP scan was performed the following day to assess for cardiac amyloidosis. However, several incidental findings were observed, including increased tracer uptake in the spleen and a large area in the left upper abdomen corresponding to an actively bleeding retroperitoneal hematoma. The presence of splenic uptake has been associated with AL cardiac amyloidosis (AL-CA).

Extracardiac Tc-99m PYP uptake should be incorporated into image interpretation to enhance diagnostic accuracy, inform treatment planning, and improve prognostic assessment. However, the clinical meaning of extracardiac uptake is not fully established, emphasizing the importance of future research to better delineate its role in identifying amyloidosis and other systemic disorders.

## Introduction

The diagnosis of transthyretin cardiac amyloidosis (ATTR-CA) remains challenging owing to a number of factors, which include the relative rarity of the disease, clinical overlap with more common diseases that result in thickening of the myocardium (i.e., hypertension, chronic renal failure, hypertrophic cardiomyopathy, and aortic stenosis), unfamiliarity with the proper diagnostic algorithm, and a perceived lack of definitive treatment [1]. Technetium-99m pyrophosphate (Tc-99m PYP) imaging plays a crucial role in diagnosing ATTR-CA [2,3]. Additionally, single-photon emission computerized tomography (SPECT) imaging is essential to differentiate true myocardial uptake from extracardiac sources, which is critical for accurate interpretation [4].

Extracardiac uptake of Tc-99m PYP is less understood and may provide insights into other underlying conditions [5]. Blood pool activity is the most common source of false-positive results on planar imaging. Because bone-avid tracers are blood pool agents, residual intravascular activity can mimic myocardial uptake, particularly in patients with reduced cardiac output or when imaging is performed early (at one hour) [4]. Bone uptake from overlying ribs or sternal structures can confound interpretation. Rib fractures overlying the heart can elevate radiotracer counts and affect heart-to-contralateral lung (H/CL) ratio calculations or mimic myocardial uptake on planar images [4]. Skeletal muscle and lung infiltration can be detected, providing additional information about extracardiac amyloid burden [4]. This report presents a rare case of extracardiac Tc-99m PYP uptake in a patient with retroperitoneal hemorrhage.

## Case presentation

A 55-year-old female patient with no history of coagulopathy or anticoagulant use presented with left flank and abdominal pain. This was associated with nausea, vomiting, fevers, and chills. Vitals upon presentation were 112/70, heart rate of 90 beats per minute, temperature of 98.9 degrees Fahrenheit, and an oxygen saturation of 98% on room air. Labs upon presentation demonstrated a leukocytosis of 17.61 K/mcL, hemoglobin of 6.3 g/dL consistent with blood loss, and a platelet count of 401 K/mcL. She had an acute kidney injury with a creatinine of 3.8 mg/dL (baseline around 1.5 mg/dL) (Table 1).

**Table 1:** Baseline laboratory values from a complete blood count and basic metabolic profile. Reference ranges with units presented on the right-sided columns.

	Value	Reference range	Units
White blood count	17.61	4.0-11.0	K/mcL
Red blood count	2.28	4.0-5.4	Million/mcL
Hemoglobin	6.3	12.0-16.0	g/dL
Platelet	410	150-400	K/mcL
Blood urea nitrogen	56	6-20	mg/dL
Creatinine	3.8	0.5-1.0	mg/dL
Sodium	136	136-145	mmol/L
Potassium	3.9	3.5-5.1	mmol/L
Chloride	106	98-107	mmol/L
CO_2_	19	22-29	mmol/L

**Figure 1:**
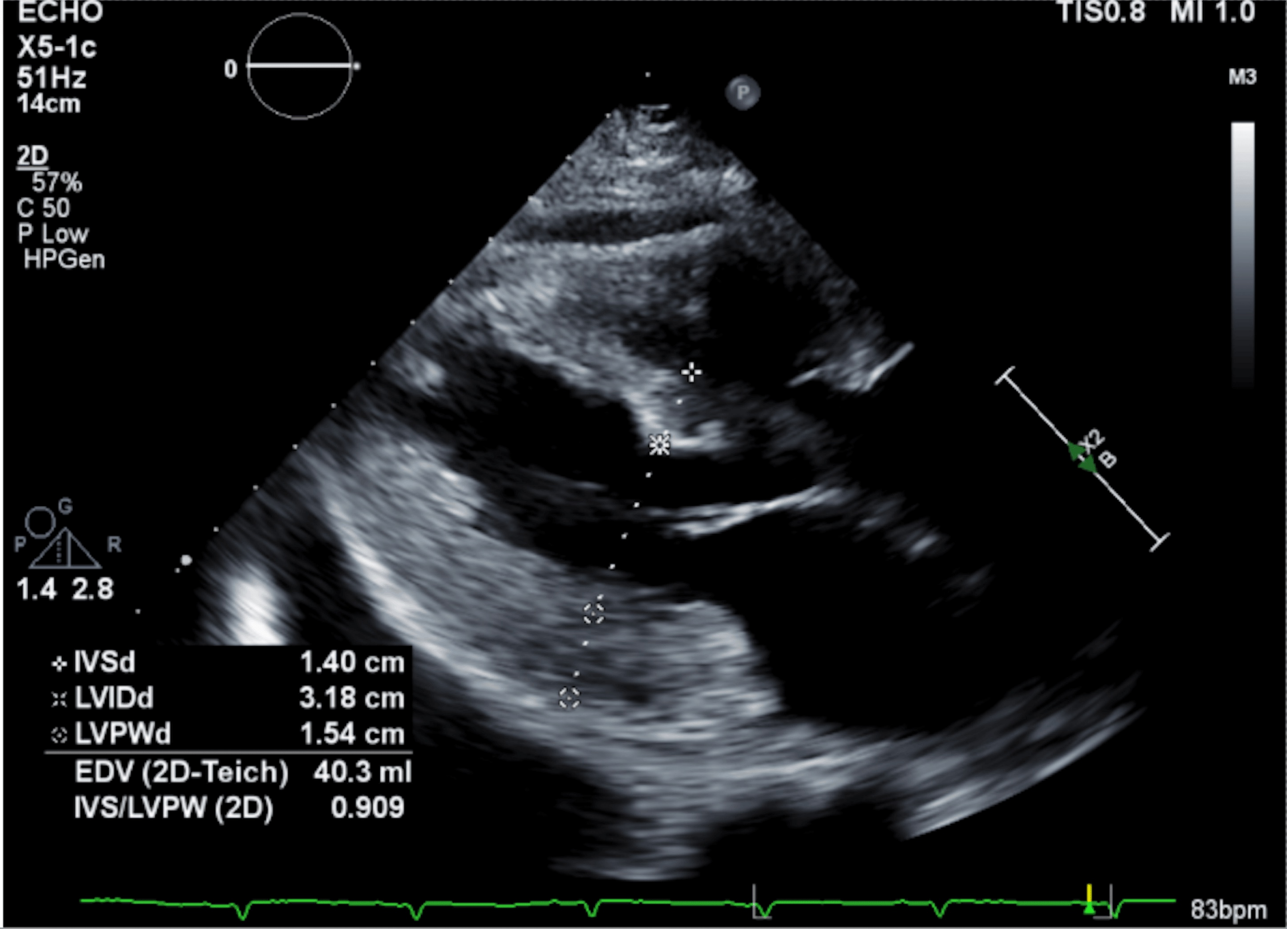
Parasternal long axis view demonstrating severely increased left ventricular wall thickness (interventricular septum measuring 1.4 cm, posterior wall measuring 1.54 cm, both at end diastole). The "speckled" appearance of the ventricle walls seen here raised concern for an infiltrative disorder.

**Figure 2:**
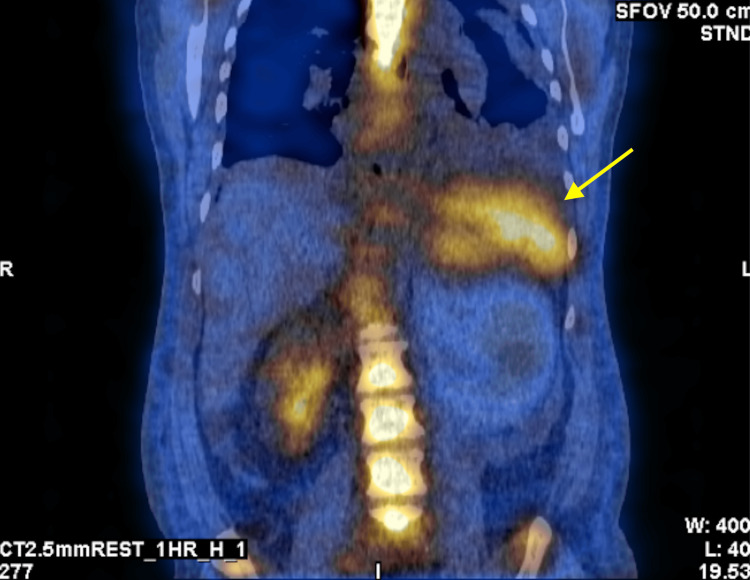
Planar SPECT images with splenic (yellow arrow) and right renal uptake of Tc-99m PYP. There is absent uptake of Tc-99m PYP in the left renal system, likely secondary to mass effect from the spleen/hemorrhage. SPECT: single-photon emission computerized tomography; Tc-99m PYP: technetium-99m pyrophosphate

Contrast tomography imaging of the abdomen and pelvis revealed a large left retroperitoneal hematoma of unknown etiology. Despite no use of anticoagulants or genetic disorders, there was no clear inciting event. An echocardiogram demonstrated cardiomyopathy with a speckled appearance and increased left ventricular wall thickness (Figure 1), raising suspicion for cardiac amyloidosis. Consequently, a Tc-99m PYP scan was performed the following day to assess for cardiac amyloidosis. The scan did not demonstrate myocardial uptake, initially ruling out ATTR-CA (Figure 2).

**Figure 3:**
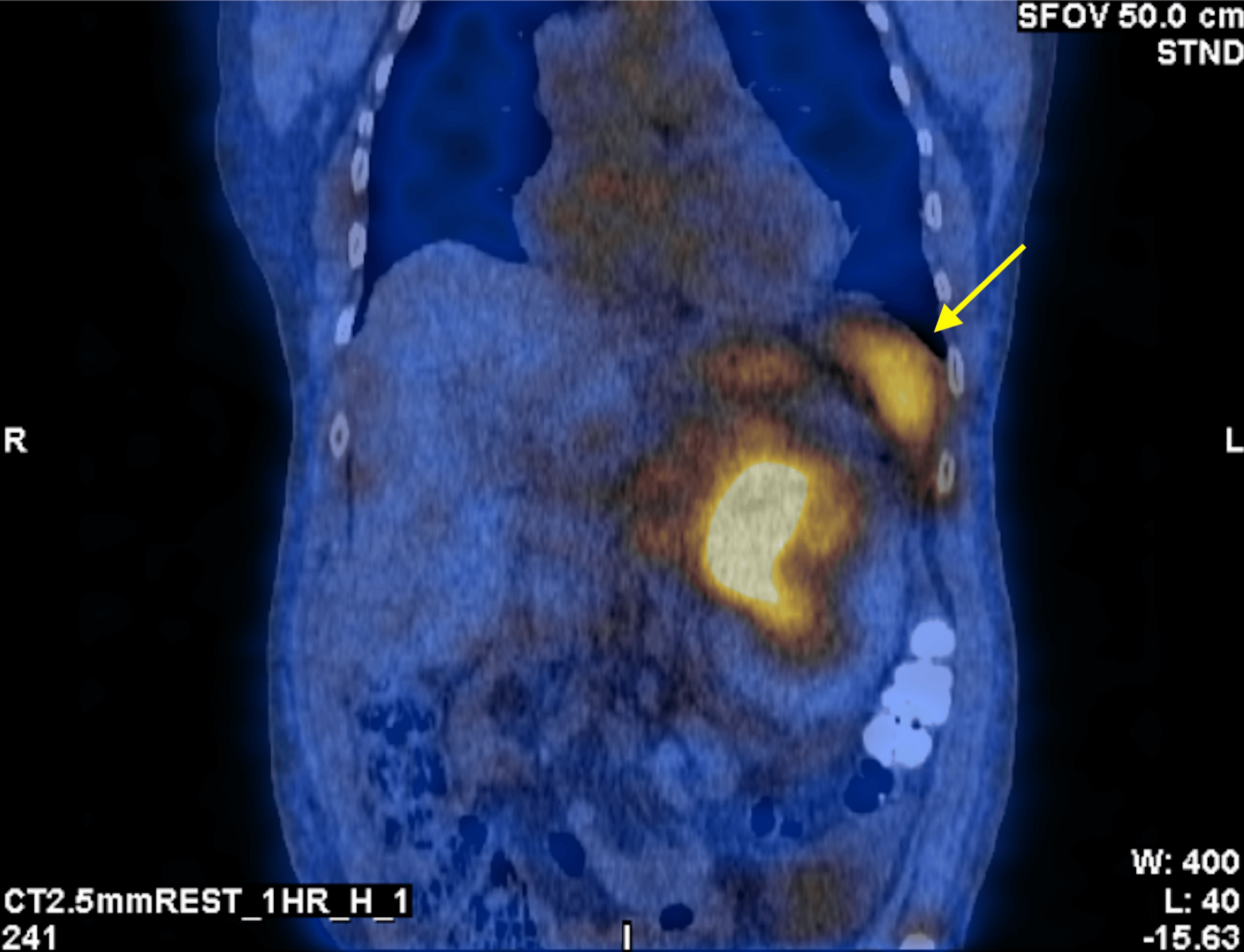
Planar SPECT images with retroperitoneal uptake of Tc-99m PYP (yellow arrow). SPECT: single-photon emission computerized tomography; Tc-99m PYP: technetium-99m pyrophosphate

**Figure 4:**
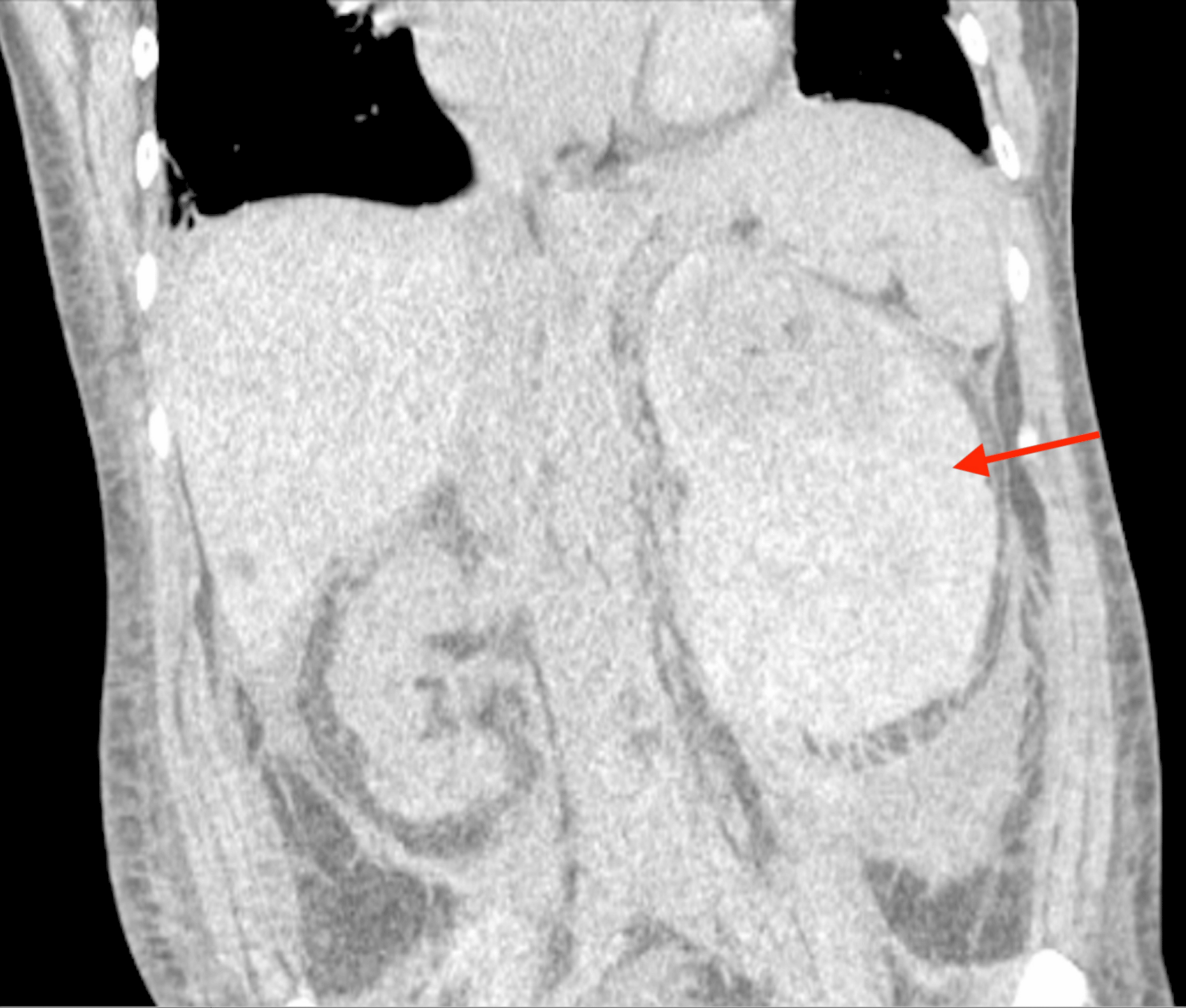
CT abdomen with notable retroperitoneal hematoma with mass effect on the left kidney (red arrow).

However, several incidental findings were observed, including increased tracer uptake in the spleen and a large area in the left upper abdomen corresponding to a retroperitoneal hematoma (Figures 3, 4).

Additionally, there was an absence of tracer uptake in the left kidney compared to the right kidney. Given ongoing hemoglobin decline and concern for active bleeding, the patient was referred to interventional radiology for potential embolization. However, urology was consulted and deemed conservative management of the retroperitoneal hemorrhage to be most appropriate while the patient remained hemodynamically stable. Due to the presence of splenic uptake, which has been associated with AL amyloidosis, the patient was seen by the oncology team. They performed serum protein electrophoresis (SPEP), urine protein electrophoresis (UPEP), and immunofixation, which were negative for AL at the time of presentation. The patient also underwent an abdominal fat pad biopsy that was negative for amyloidosis. However, the oncology teams continued to stress that this negative fat pad biopsy did not fully rule out amyloidosis. Unfortunately, shared decision making between nephrology and oncology ruled a renal biopsy too high risk after presenting with a retroperitoneal hemorrhage initially. The patient stabilized and was discharged with close outpatient follow-up.

## Discussion

This case demonstrates uncommon manifestations of extracardiac Tc-99m PYP uptake that provided critical diagnostic and management insights. Extracardiac uptake patterns remain incompletely characterized and warrant careful evaluation. Previous studies have shown that 29% of patients with ATTR-CA or AL-CA undergoing Tc-99m scintigraphy exhibit extracardiac uptake [3]. Although commonly reported sites of uptake include the pulmonary and gastrointestinal tracts, as well as subcutaneous tissues, this case highlights incidental extracardiac Tc-99m PYP uptake in both a perinephric hematoma, which was critical in guiding the management of retroperitoneal hemorrhage due to ongoing concern for active bleeding.

Increased tracer uptake in the liver and spleen has been observed in cases of AL-CA [6]. Splenic uptake on Tc-99m PYP imaging is an exceptionally rare finding that has been documented primarily in patients with ATTR-CA, reflecting systemic amyloid deposition [7]. There was no clear etiology of the retroperitoneal bleed, but possible differentials include a vascular malformation, vasculitis, or genetic hematologic disorders predisposing the patient to bleeding. While the absence of myocardial uptake ruled out ATTR-CA in this patient, splenic uptake raised concerns for systemic AL amyloidosis, necessitating close oncologic follow-up.

While Tc-99m PYP scintigraphy has gained prominence for cardiac amyloidosis diagnosis, this radiotracer has established clinical applications beyond amyloid imaging. Tc-99m PYP was originally developed and FDA-approved for skeletal imaging to demonstrate areas of altered osteogenesis, cardiac imaging as an adjunct in acute myocardial infarction diagnosis, gated cardiac blood pool imaging, and detection of gastrointestinal bleeding [8]. In this case, the incidental detection of asymmetric renal uptake proved clinically significant. Tc-99m PYP undergoes renal excretion, with approximately 40% of the administered dose excreted in urine within 24 hours under normal physiological conditions [8,9]. Additionally, Tc-99m PYP demonstrates homogeneous uptake in the kidneys, which is expected under normal physiological conditions [10]. The observed reduction in left kidney uptake, attributed to mass effect and impaired renal function from the large perinephric hematoma, provided crucial information regarding ongoing hemorrhage risk and guided clinical management decisions.

This case illustrates that a comprehensive interpretation of Tc-99m PYP scans should extend beyond cardiac assessment. Systematic evaluation of extracardiac uptake patterns can identify (1) systemic amyloid involvement, suggesting AL rather than ATTR disease, (2) acute processes such as active hemorrhage or myocardial infarction, and (3) anatomic abnormalities affecting normal tracer biodistribution.

## Conclusions

We reported a rare case of extracardiac Tc-99m PYP uptake in a patient with retroperitoneal hemorrhage. Extracardiac Tc-99m PYP uptake should be incorporated into image interpretation to enhance diagnostic accuracy, inform clinical decision-making, and improve prognostic assessment. This further emphasizes the importance of future research to better delineate its role in identifying amyloidosis and other systemic disorders.
